# Prevalence and predictors of exclusive breastfeeding among women in Kilimanjaro region, Northern Tanzania: a population based cross-sectional study

**DOI:** 10.1186/1746-4358-8-12

**Published:** 2013-10-09

**Authors:** Melina Mgongo, Mary V Mosha, Jacqueline G Uriyo, Sia E Msuya, Babill Stray-Pedersen

**Affiliations:** 1 Department of Community Health, Kilimanjaro Christian Medical University College, Box 2240, Moshi, Tanzania; 2 Better Health for African Mother and Child (BHAMC), Box 8418, Moshi, Tanzania; 3 Division of Women and Children, Oslo University Hospital Rikshospitalet and University of Oslo, Oslo, Norway; 4 Kilimanjaro Christian Medical Centre, Po Box 3010, Moshi, Tanzania

**Keywords:** Exclusive breastfeeding, Prevalence, Predictors, Alcohol, Health worker advice, Tanzania

## Abstract

**Background:**

Exclusive breastfeeding (EBF) is a simple and cost-effective intervention to improve child health and survival. Effective EBF has been estimated to avert 13% - 15% of under-five mortality and contribute to reduce mother to child transmission of HIV. The prevalence of EBF for infant less than six months is low in most developing countries, including Tanzania (50%). While the Tanzania Demographic Health Survey collects information on overall EBF prevalence, it does not evaluate factors influencing EBF. The aim of this paper was to determine the prevalence and predictors of exclusive breastfeeding in urban and rural areas in Kilimanjaro region.

**Methods:**

A population-based cross-sectional study was conducted between June 2010 to March 2011 among women with infants aged 6–12 months in Kilimanjaro. Multi-stage proportionate to size sampling was used to select participants from all the seven districts of the region. A standardized questionnaire was used to collect socio-demographic, reproductive, alcohol intake, breastfeeding patterns and nutritional data during the interviews. Estimation on EBF was based on recall since birth. Multivariable logistic regression was used to obtain independent predictors of EBF.

**Results:**

A total of 624 women participated, 77% (483) from rural areas. The prevalence of EBF up to six months in Kilimanjaro region was 20.7%, without significant differences in the prevalence of EBF up to six months between urban (22.7%) and rural areas (20.1%); (OR = 0.7, 95% CI 0.5,1.4).

In multivariable analysis, advice on breastfeeding after delivery (Adjusted odds ratio, AOR = 2.6, 95% CI 1.5, 4.6) was positively associated with EBF up to six months. Compared to married/cohabiting and those who do not take alcohol, single mothers (AOR = 0.4, 95% CI 0.2, 0.9) and mothers who drank alcohol (AOR = 0.4, 95% CI 0.3, 0.7) had less odds to practice EBF up to six months.

**Conclusion:**

Prevalence of EBF up to six months is still low in Kilimanjaro, lower than the national coverage of 50%. Strengthening of EBF counseling in all reproductive and child health clinics especially during antenatal and postnatal periods may help to improve EBF rates.

## Background

Exclusive breastfeeding (EBF) is a simple, cheap and cost effective intervention in reducing child mortality and morbidity in low income countries [1,2]. EBF means an infant receives breast milk from his or her mother or expressed breast milk or a wet nurse for the first six months of life and no other solids/semisolids are given with exception of vitamins, mineral supplements or medicine [3]. It has been estimated that EBF coverage of 90% will help to improve child survival [1]. EBF is recommended during the first six months of infants’ life because it confers many nutritional and health benefits to the child [3]. If well implemented it has been estimated to avert 15% of child deaths and in high HIV prevalence settings, it has been estimated to avert 13% of child deaths [1]. Exclusively breastfed children have shown to have lower risk of gastrointestinal infections and acute respiratory infection compared to children who were not exclusively breastfed [4-7]. EBF has also shown to reduce the rate of mothers to child transmission of HIV compared to mixed feeding practices [8].

WHO reported an overall prevalence of EBF of 36%, the highest rates of EBF were found in East Asia/Pacific (43%) and the lowest rates of EBF in West/Central Africa (20%) [9,10]. In Sub Saharan Africa where there is high rates of mother to child HIV transmission, malnutrition, infant and child mortality rates, the overall prevalence of EBF was 33% [11]. Tanzania, a country with high infants’ and child mortality rates (51 and 81 per 1000 live births respectively), and high rate of stunting for children under age of 5 (42%), has high suboptimal breastfeeding practices and low prevalence of EBF for infants under 6 months (50%) [12]. According to the countdown report of 2012, Tanzania is among the countries which have made insufficient progress to meet the Millennium development goal 4. The target is to reduce under five mortality to 56/1000 live birth by 2015 (MDG 4) [13].

Information on local factors that influence EBF is vital in guiding strategies to improve EBF trend. The factors influencing EBF have shown to vary from country to country and within countries. Employment status, urban/rural differences, marital status, knowledge on breastfeeding, education status, place of delivery, HIV status, advice on breastfeeding, ant-natal care clinic (ANC) attendance and type of delivery have shown to have an influence on EBF [13-17]. While the Tanzania Demographic and Health Surveys (TDHS) collects data on national prevalence of EBF, it does not generate regional rates, nor does it provide detailed information on predictors of EBF. Further many studies on breastfeeding in Tanzania have only been concentrated in HIV positive women or prevention of mother to child transmission (PMTCT) of HIV [18,19]. Among few studies on predictors of EBF conducted on general population of women, were either done in the late 90s or based in the urban settings [14,17,20]. This paper aimed to determine the prevalence and predictors of EBF in urban and rural areas in Kilimanjaro region, northern Tanzania.

## Methods

### Study design and area

This population based cross-sectional study was part of a larger study that was conducted from June 2010 to March 2011 involving mothers with children aged 0–36 months in all 7 districts of Kilimanjaro region i.e. Same, Mwanga, Rombo, Moshi rural, Moshi urban, Siha and Hai. The larger study was conducted to determine the developmental norms of children in Kilimanjaro region and investigated local risk factors for poor child development [21]. The analysis for this part of the study was limited to women who had ever breastfed, reported breastfeeding history of the infant and with infants aged 6–12 months at the time of interviews. The prevalence and predictors of EBF in Kilimanjaro were investigated as one of the nutritional component for child development.

The region is one of the 24 administrative regions of Tanzania Mainland and is situated in the Northern part of the country. The region has a population of 1,376,702, of whom 335,790 are women of reproductive age (15–49 years) and 42,661 infants. The region has a population annual growth rate of 1.6%. The large population (75%) lives in the rural area and depend on agriculture and livestock keeping. Other economic activities include tourism, manufacturing, fishing and in a small scale beekeeping [22].

The region has 18 hospitals, 41 health centres and 326 dispensaries. These are either owned by the government, religious or private sectors. All hospitals and health centres provide reproductive and child health care including ANC, deliveries, postnatal care, family planning, immunization, growth monitoring and preventive activities.

According to the recent statistics on Kilimanjaro region from TDHS, attendance to reproductive health services is high; ANC coverage is 100%, delivery in the health facility 86.7% and postnatal care 48.4% compared to the national level of 96%, 50% and 30% respectively. In addition, 55.2% of infants are breastfed within one hour of birth, 92.7% within 24 hours after birth, and the median duration of EBF is 2.4 months [12].

### Sampling procedure

Village and street by single age population data from the 2002 census with 2009 projections were obtained from the Kilimanjaro regional bureau of statistics office in April 2009. A multistage sampling design was used. To start with, 0–36 month’s population for each village/street were listed with a column showing its cumulative population to be used as a sampling frame. The sampling interval was then calculated: Total population of 0–36 month olds divided by the required number of clusters i.e. 50. A random number between 0 and 1 was generated from a computer and the starting point for selection of the first cluster was determined by multiplying the random number with the sampling interval. The subsequent cluster was located by adding the sample interval to the previous number until the 50th cluster was selected. Compact segment sampling was used to select households within clusters. The selected clusters i.e. enumeration areas was mapped into segment with an approximately equal population. The number of segments was equal to the total population of 0–36 month olds divided by the cluster size i.e.50. Each segment included 50 children. All segments were assigned a number on pieces of paper and one was randomly picked. Within the selected segment, the study team visited all the households until 50 children whose parents consented were examined. If all the households in the segments had been surveyed and less than 50 children were available, a second segment was randomly selected. The members of the households in the selected segments were informed to be available on the day of the survey.

### Interviews

Structured questionnaires were used to collect the information from mothers. During the interviews information collected were: socio demographic characteristics (age, marital status, education, employment, alcohol intake), reproductive information (parity, place of delivery, assistance during delivery, ANC attendance, advice on breastfeeding during ANC and postnatal period), HIV status of the mother using a verbal report and information about the partner age and education. Information on child nutritional history (breastfeeding initiation, duration of EBF in days, breastfeeding duration and frequency, time for the introduction of liquid/semisolids/solids in days), anthropometric measurements (weight in kg, height in cm) was taken. Height was measured prone using length stadiometer. Weights of undressed children were taken on a SECA Digital Scale. Height-for-Age (HAZ) and Weight-for-Age (WAZ) scores were generated using the WHO software for assessing growth and development [23]. All anthropometric measures were taken by two trained assistants.

**Figure 1:**
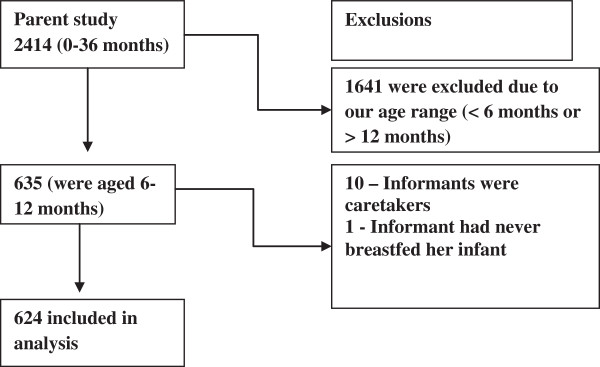
Illustration of mother-infant pairs extracted from the main study.

There were 635 infants aged 6–12 months. However the analysis included 624 mothers who had ever breastfed and reported on breastfeeding history of the infant, see Figure 1.

### Data processing and analysis

Data were analyzed using Predictive Analytical Soft Ware (PASW) version 18. Exclusive breastfeeding was measured using report of exclusive breastfeeding (duration of EBF) from the mother and the introduction of liquids/semisolids food. Mothers who did not introduce liquids/semisolids to their infants up to six months were categorized as practiced exclusive breastfeeding. Descriptive statistics were used to summarize the data and difference between groups was compared using Chi squared test and Fishers exact test as appropriate. The odds ratio (OR) and their 95% confidence interval (CI) were obtained from to assess the strength of association between several independent predictors and EBF (dependent variable). All predictors with p value of < 0.05 in the univariate logistic regression were included in the multivariable analysis model. The multivariable logistic regression using forward selection of variable was performed to get the independent predictors of EBF. Though area of residence was not significant predictor in the univariate model it was included in the multivariable model because it was an important exposure.

### Ethics

Ethical clearance for the parent study was obtained from National Institute for Medical Research (NIMR) Tanzania (certificate number 938). Permission to use the current data was sought from Kilimanjaro Christian Medical University College (KCMUCo) research and ethics review committee (Certificate number.461). Permission to conduct the study was also sought from each respective village/ward governments. All the women who participated in the study gave written informed consent.

## Results

In total 624 mother-infant pairs who met the inclusion criteria were included in analysis. Of these 77.3% (n = 483) were from rural area. The age of the participating mothers ranged 15–45 years with mean of 27.98 years (standard deviation (SD) of 7.1 years). Majority of women were married or cohabiting 83% (n = 517), had completed primary school education 78% (n = 468), and had less than 4 children 83% (n = 516). Table 1 shows the demographic characteristics of the participants by rural and urban residence.

**Table 1:** Socio-demographic characteristics of the respondents by residence

**Variable (mean ± SD or %)**	**Urban**	**Rural**	**Total**
	**n = 141**	**n = 483**	**n = 624**
**Mothers**			
Mothers age	26.2 ± 5.3	28.5 ± 7.5	28 ± 7.1
Mothers years of education	7.7 ± 2.0	7.0 ± 2.2	7.1 ± 2.2
Number of children	2.0 ± 1.1	3.1 ± 2.1	2.8 ± 1.9
Alcohol intake (n = 620)	19.1% (27)	44.7% (216)	38.9% (243)
Partners**			
Fathers age (n = 590)	31.9 ± 6.5	35.0 ± 11.7	34 ± 10.5
Years of education (n = 605)	8.4 ± 2.6	7.8 ± 2.2	7.9 ± 2.4

**Variable with missing information.

*SD* standard deviation.

The mean age of the infants was 8.75 (SD = 1.95) months and the median was 9 months. There was no significant difference in the mean age and sex of the urban and rural infants.

The prevalence of EBF for infants up to six months in Kilimanjaro was 20.7% (n = 129). At one month the prevalence of EBF was 88.3% (n = 551) and at three months the prevalence was 65.5% (n = 409). There were no significant differences in the prevalence of EBF up to six months between urban 22.7% (n = 32) and rural areas 20.1% (n = 97), p value = 0.56. Table 2 includes further results of EBF prevalence by area of residence i.e. urban and rural.

**Table 2:** Prevalence of exclusive breastfeeding by age among 624 women in rural and urban areas in Kilimanjaro

**Age in months**	**Frequency**	**%**	**p-value**
**At one month**			
Urban	126	89.4	0.77
Rural	425	88.0	
**At 3 months**			
Urban	102	72.3	0.06
Rural	307	63.6	
**At 5 months**			
Urban	41	29.1	0.83
Rural	135	28.0	

Seventy nine percent of the mothers (n = 493) initiated breastfeeding within one hour after delivery. Mothers who were assisted by the skilled health staff during delivery were less likely to initiate breastfeeding within one hour 78.2% (n = 445) compared to those assisted by traditional birth attendants and family members 94.0% (n = 47), p-value = < 0.01. Ninety one percent of mothers were still breastfeeding their infants during the study period. One percent of the mothers gave prelacteal feed to their infants. Twelve percent of the mothers (n = 80) gave their infant water during the first month of life at a median age of one month. At 3 months, 35% of the mothers gave their infants mtori (mashed cooked banana plus beef soup and/or other vegetables) and smooth porridge.

In the analysis for predictors of EBF up to six months, there were no urban and rural differences for all the major predictors evaluated (data not shown). Analysis of predictors was therefore performed without stratifying for place of residence. In the univariable logistic regression, single mothers had decreased odds of exclusive breastfeeding compared to married/cohabiting mothers [OR 0.4; (95% CI: 0.2, 0.8)]. Mothers drinking alcohol had decreased odds of exclusively breastfeeding compared to those who were not drinking alcohol; [OR 0.5; (95% CI: 0.3, 0.7)], see Table 3. Women with partners who had secondary school had decreased odds of practicing EBF compared to women with partners who had primary education, [OR = 0.4; (95% CI: 0.1, 0.9)]. On the other hand, the odds of exclusively breastfeeding the infants up to 6 months was 3 times higher among mothers who got advice on breastfeeding during ANC attendance and after delivery compared to mothers who did not get advice during antenatal or postnatal period. Other factors like age, education, occupation, parity, type of delivery, HIV status, sex of the child and nutritional status of the child were assessed but not associated with EBF up to six months in Kilimanjaro (Table 3).

**Table 3:** Univariable and multivariable logistic regression of predictors of exclusive breastfeeding in Kilimanjaro region (n = 624)

**Variable**	**n**	**n (%) EBF**	**COR**	**95% CI**	**AOR**^**∞**^	**95% CI**
**Mothers information**						
**Age in years**						
Less than 20	44	9 (20.5)	Reference			
20-34	462	95 (20.6)	1	0.5, 2.2	-	-
35+	118	25 (21.2)	1.1	0.4, 2.5	-	-
**Marital status**						
Married/cohabiting	517	129 (22.8)	Reference			
Single	107	11 (10.3)	0.4	0.2, 0.8	0.4	0.2, 0.9
**Employment**						
No	136	27 (19.9)	Reference			
Yes	488	102 (20.9)	1.1	0.7, 1.7	-	-
**Occupation**						
Unemployed	142	27 (19.0)	Reference			
Agriculture	257	54 (21.0)	1.1	0.7, 1.9	-	-
Small scale business	154	38 (24.7)	1.4	0.8, 2.4	-	-
Casual/unskilled	35	4 (14.3)	0.7	0.3, 2.0	-	-
Skilled/professional	36	5 (13.9)	0.7	0.2, 1.9	-	-
**Years of education**						
Primary incomplete	44	10 (22.7)	Reference			
Primary complete	468	98 (20.9)	0.9	0.4, 1.9	-	-
Secondary school and above	112	21 (18.8)	0.8	0.3, 1.8	-	-
Alcohol intake (n = 620)**						
No	377	95 (25.2)	Reference			
Yes	243	33 (13.6)	0.5	0.3, 0.7	0.4	0.2, 0.6
**Number of children**						
One child	192	39 (20.3)	Reference			
2-4 children	324	69 (21.3)	1.1	0.7, 1.7	-	-
5 + children	108	21(19.4)	0.9	0.5, 1.7	-	-
**Type of delivery**						
Spontaneous vaginal delivery	579	124 (21.4)	Reference			
Caesarean section	45	5 (11.1)	0.5	0.2, 1.2	-	-
HIV results (n = 605)**						
HIV-	584	121 (20.7)	Reference			
HIV+	19	3 (15.8)	0.7	0.2, 2.5		-
Didn’t provide the results	2	1 (50.0)	3.8	0.2, 61.6		-
Advised on breastfeeding during ANC (605)**						
No	175	19 (10.9)	Reference			
Yes	430	108 (24.1)	2.6	1.6, 4.7	1.6	0.9, 3.0

**- variables with missing information.

∞- variables included in the model: marital status, advice on breastfeeding during ANC and after delivery, child age, fathers level of education, area of residence.

*ANC* antenatal care.

*AOR* adjusted odds ratio.

*BMI* body mass index.

*CI* confidence interval.

*COR* crude odds ratio.

*EBF* exclusive breastfeeding.

In multivariable logistic regression, advice on breastfeeding after delivery, [adjusted odds ratio (AOR) = 2.6; (95% CI: 1.5, 4.6)], single mothers [AOR = 0.4; (95% CI: 0.2, 0.9)] and alcohol intake [AOR = 0.4; (95% CI: 0.3, 0.7)] remained associated with EBF up to 6 months. Other factors like advice on breastfeeding during ANC visit, age of the child, area of residence, partner’s education were assessed but not associated with EBF up to six months in Kilimanjaro (Table 3).

## Discussion

Results showed a low prevalence of EBF practice in Kilimanjaro region. There was no significant difference in EBF rates between urban and rural areas. Advice on breastfeeding after delivery was an important factor for mothers to practice EBF. Single mothers and those who drank alcohol had decreased odds to practice EBF for six months.

High prevalence of EBF was found to be at one month in both urban and rural areas. The general prevalence of EBF in Kilimanjaro was 88.1% at one month, 65.5% at three months and 20.7% up to six months. Declining trend in EBF prevalence have also been shown by the TDHS where 81%, 51% and 23% of the infants at < 2 months, 2–3 months and 4–5 months respectively were exclusively breastfed [12]. In Kilimanjaro region, there is a traditional practice where mothers are relieved from their daily chores in the first three months after delivery. During this period mother in laws or grandmothers have to feed lactating mothers with special diet believed to enhance milk production. This could explain the higher rates of EBF at the first three months after delivery.

The prevalence of EBF is low compared to the national rate of 50% and is far from the estimated coverage of 90% [1]. In our study we have used recall since birth to estimate EBF prevalence this could have contributed our low prevalence compared to TDHS which used 24 hours recall. Study has shown that 24 hour recall tends to overestimate the EBF rates; for example in Uganda the EBF prevalence was 7% at three months when using recall since birth and 81% by using 24 hours recall [24]. Differences in study designs could explain the differences in prevalence between and within countries [25].

The introduction of prelacteal feed is discouraged because it limits the frequency of suckling and exposes the infant to risks of infections [12,26]. About one percent of the mothers gave prelacteal feeds (mainly water) to their infants. The rate of prelacteal feeding was low compared to the national report of 31% [12]. In Kigoma higher rates (14%) of giving prelacteal feeds to the infants were also reported [14]. We might have got low proportion of prelacteal feeding compared to other researchers due to long recall bias. Or it might be due to socio desirability bias as the mothers might have reported the correct answer but not the real practice.

Complementary feeding started early; at the age of three months 35% of the mothers gave their infants smooth porridge and *mtori*. Most of the women in this study (about 80%) were engaged in income generating activity both in formal and informal sector. It may be that women are forced to introduce other foods before resuming work as maternity leave is 84 days in Tanzania. Another explanation may be that either women have little knowledge on the WHO recommendations of infants feeding, or do not believe EBF alone is enough for infants’ growth in the first six months [17,24,27,28]. A qualitative study is needed to give an insight about women’s attitudes and beliefs about EBF in the region, given most of the demographic characteristics were not associated with EBF.

Mothers who drank alcohol had decreased odds to exclusively breastfeed their infants for six months compared to non drinkers. In this study 39% of lactating mothers were drinking alcohol. Studies in Brazil and Australia have shown alcohol intake was associated with reduced duration of EBF [29,30]. It may be mothers who drink tend to spend less time with their infants and tend to leave them in care of others, who are forced to feed them with other foods when mothers are not around. Alcohol intake has also shown to be associated with other negative maternal and child health indicators like HIV and poor attendance for child immunization [31,32]. In this setting the prevalence of alcohol intake among women ranges from 31% -39% [32,33]. Traditionally, alcohol is used widely in Kilimanjaro, in various occasions or celebration such as weddings, burials, and payment of other disciplinary fines. There is a need to have community programs that will address the effects of alcohol intake in pregnant and lactating women. They should discourage women from alcohol use during pregnancy or breastfeeding as it negatively affects newborns, EBF, and general infant growth [30,31].

Single mothers had significantly lower prevalence of EBF for six months compared to cohabiting or married women. It may be single mothers lack social support to continue practicing EBF as they have to earn for the family. Reports from Canada, Norway and Uganda showed that mothers living with their partners were more likely to exclusively breastfeed their infants for six months [16,24,34] while in Ethiopia single mothers were two times more likely to exclusively breastfeed their infants compared to married ones [35].

Advice on breastfeeding after delivery was a positive factor for mothers to exclusively breastfeed their infants for the first six months of infant’s life. Hospital delivery on its own did not increase the odds of EBF in this study, differing from others [17,36]. Rather it is the breastfeeding counselling given by health providers to women during pregnancy, immediately after delivery or when they bring their children to the clinic for immunization. Studies from Malawi, Zambia and Morogoro, Tanzania showed that, women tend to believe and follow what they are taught by the health professionals and their advice was shown to positively influence EBF practice [17,36,37].

A difference in EBF rates for urban and rural mothers which was observed in other settings was not observed in this study [17,38]. In those studies women from the rural areas were more likely to give prelacteal feeds and mix fed earlier compared to urban mothers. It may be that, in Kilimanjaro mothers in rural and urban areas are equally exposed to information/media, have equal access to health care and have similar demographic or reproductive health factors. There is a need for further studies to explore whether it is knowledge, cultural factors, or simply myths which affects EBF in the region.

The study had the following limitations. Recall bias could have affected the results. We studied mothers with infants aged 6–12 months and asked feeding practices of the infants when they were less than six months. Mothers might not remember the exact time when weaning started as the information collected was based on the mother’s report of breastfeeding history. Estimating EBF with a cross-sectional study using recall since birth may be difficult and inaccurate; a cohort study would have given reliable estimates as mothers with infants would be followed after delivery and their records of breastfeeding practices could be recorded prospectively. Despite the limitations, this study has its strengths. Women with infants who participated in the study were randomly selected from the whole region. Second the data for this study was collected from the whole of Kilimanjaro region and participation rate was high. Therefore, the results on breastfeeding practices observed in this study can be generalized to the whole region.

## Conclusion

Prevalence of EBF for infants up to six months in Kilimanjaro was low (20.7%), with lack of variation between urban and rural areas. The differences in methodology estimating EBF prevalence could have contributed to our low prevalence compared to previous Tanzanian studies. Advice on breastfeeding after delivery, single mothers and alcohol intake influenced the EBF practice in the region. Since breastfeeding advice by providers increased the EBF prevalence, integration and/or strengthening of nutrition counseling at different reproductive and child health (RCH) clinics in the region is needed. We advice health education on the negative effects of alcohol needs to be given to lactating mothers at the RCH and the community at large, as it has shown to be associated with reduced duration of exclusive breastfeeding. Further, a study to explore cultural factors that influence EBF is needed in the region.

## Competing interests

The authors declare that they have no competing interests.

## Authors’ contributions

MM, JGU, SEM, BSP contributed to the design of the study. MM and JGU collected the data. MM, MVM and JGU analyzed the data.MM and SEM interpreted the results. MM prepared the manuscript and all the other authors reviewed the manuscript before submission. All authors read and approved the final manuscript.
